# Supplementary material for: Semantic and cognitive tools to aid statistical science: replace confidence and significance by compatibility and surprise
Source: BMC Med Res Methodol. 2020 Sep 30;20:244. doi: 10.1186/s12874-020-01105-9 (PMC7528258; doi:10.1186/s12874-020-01105-9)
Supplement: Supplementary file 1 — Additional file 1: Appendix. Technical details for computations of figures and tables. [file 12874_2020_1105_MOESM1_ESM.pdf]

Appendix: Technical details for computations of figures and tables.

Table 1 shows relations under the standard 1 degree-of-freedom (df) χ^2 approximation for the LR statistic when **H** is a hypothesis that a parameter equals a specific value, e.g., for the hypothesis that a hazard ratio HR equals the number r, **H**: HR = r. For normal (Gaussian) data these relations are exact and the LR statistic reduces to squared Z-score for the hypothesis [1]. The *S*-value and LR statistic track each other rather closely although the latter increases more rapidly. Their relation reflects that, under the test model and the standard approximations, the *P*-value is uniform and hence the *S*-value is unit-exponential, which is half a 2 df χ^2 [2] and hence has a heavier right tail than the 1 df LR statistic; specifically, with x = ln(r), the ratio of densities for the 2 df and 1 df χ^2 is proportional to $x^{½}$.

For **Table 2** and the figures, statistics were computed from the approximate normal distribution used for the CIs in Brown et al. [3], in which the log-hazard ratio ln(HR) is estimated to have mean m = ln(1.61) and standard deviation d = ln(2.59/0.997)/2(1.96). The *P*-value for **H**: HR = r is then derived from the normal score Z = ln(1.61/r)/d, and the LR statistic and MLR are approximated by Z^2 and $\exp(Z^2/2)$. For contrast to the *P*-graph in **Fig. 2**, **Figure S1** shows the relative likelihood function, 1/MLR, produced from the Brown et al. HDPS results, taking the maximum as the reference point so that the graph extends from 0 to 1. It may be noticed that this function appears proportional a posterior probability density for ln(HR), but this proportionality holds only under very special conditions. For contrast to the *S*-graph in **Fig. 3**, **Figure S2** shows the corresponding deviance function 2ln(MLR).

References

1. Cummings P. Analysis of Incidence Rates. CRC Press; 2019. https://doi.org/10.1201/9780429055713.

2. Fisher RA. Statistical methods for research workers. Oliver and Boyd: Edinburgh; 1925. https://books.google.com/books?id=GmNAAAAAIAAJ&q.

3. Brown HK, Ray JG, Wilton AS, Lunsky Y, Gomes T, Vigod SN. Association between serotonergic antidepressant use during pregnancy and autism spectrum disorder in children. J Am Med Assoc. 2017;317:1544–52.
