# Supplementary figures and images for: Semantic and cognitive tools to aid statistical science: replace confidence and significance by compatibility and surprise
Source: BMC Med Res Methodol. 2020 Sep 30;20:244. doi: 10.1186/s12874-020-01105-9 (PMC7528258; doi:10.1186/s12874-020-01105-9)

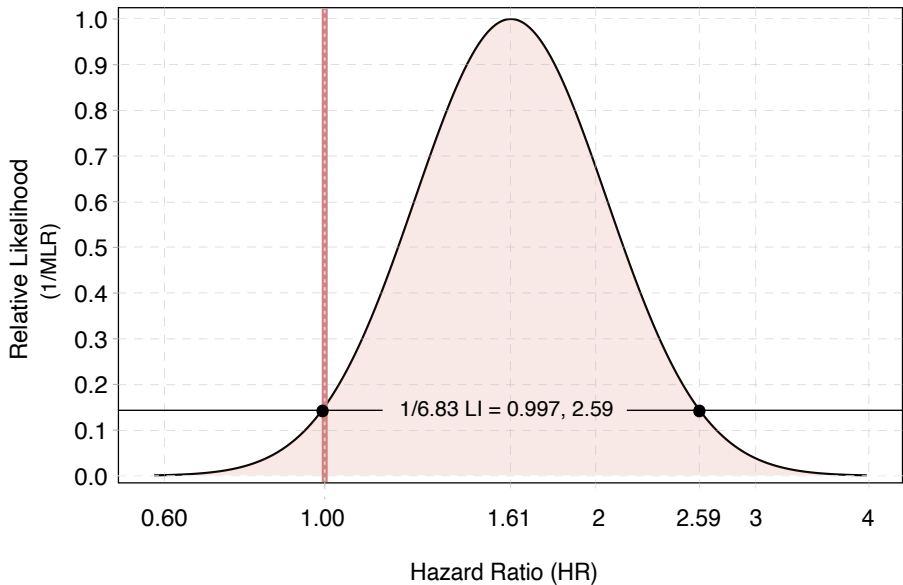

Supplement: Supplementary file 3 — Additional file 3: Figure S1. Relative likelihoods for a range of hazard ratios. A relative likelihood function that corresponds to Fig. 2, the P-value function. Also plotted is the 1/6.83 likelihood interval (LI), which corresponds to the 95% compatibility interval. Computed from results in Brown et al. [34]. MLR = Maximum-Likelihood Ratio. HR = 1 represents no association. [file 12874_2020_1105_MOESM3_ESM.pdf]

Deviance Statistic

$2\ln(\text{MLR})$

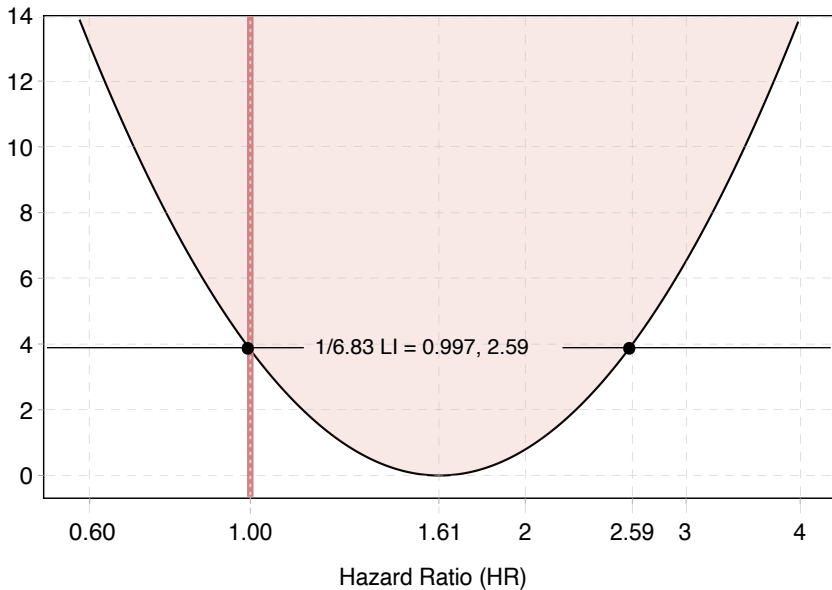

Supplement: Supplementary file 4 — Additional file 4: Figure S2. Deviance statistics for a range of hazard ratios. A deviance function, which corresponds to Fig. 3, the S-value function. Also plotted is the likelihood interval (LI), which corresponds to the 95% compatibility interval. Computed from results in Brown et al. [34]. MLR = Maximum-Likelihood Ratio. HR = 1 represents no association. [file 12874_2020_1105_MOESM4_ESM.pdf]
